# Mechanistic insights in the role of trehalose transporter in metabolic homeostasis in response to dietary trehalose

**DOI:** 10.1093/g3journal/jkaf303

**Published:** 2025-12-30

**Authors:** Bhagyashri Y Chaudhari, Vikram J Nichit, Vitthal T Barvkar, Rakesh S Joshi

**Affiliations:** Biochemical Sciences Division, CSIR-National Chemical Laboratory, Dr. Homi Bhabha Road, Pune, Maharashtra 411008, India; Academy of Scientific and Innovative Research (AcSIR), Ghaziabad, Uttar Pradesh 201002, India; Biochemical Sciences Division, CSIR-National Chemical Laboratory, Dr. Homi Bhabha Road, Pune, Maharashtra 411008, India; Academy of Scientific and Innovative Research (AcSIR), Ghaziabad, Uttar Pradesh 201002, India; Department of Botany, Savitribai Phule Pune University, Pune, Maharashtra 411007, India; Biochemical Sciences Division, CSIR-National Chemical Laboratory, Dr. Homi Bhabha Road, Pune, Maharashtra 411008, India; Academy of Scientific and Innovative Research (AcSIR), Ghaziabad, Uttar Pradesh 201002, India

**Keywords:** trehalose, sugar uptake, metabolism, transporters, *Helicoverpa armigera*, gut

## Abstract

Trehalose is the major sugar in insect hemolymph and plays a diverse role. Its level is regulated by the dynamics of biosynthesis and distribution by sugar transporters. The metabolic balance between trehalose synthesis and uptake remains poorly understood, despite its critical role in homeostasis. Here, we examined the role of the putative gut-specific sugar transporter, *HaST46*, in regulating trehalose levels in *Helicoverpa armigera*, a model Lepidopteran pest. Analysis of publicly available transcriptomics, proteomics data, and qRT-PCR study suggests that HaST46 showed localization in the posterior midgut and its expression alters in response to dietary contents. The liquid chromatography-mass spectrometry (LC-MS) analysis of HaST46 overexpressing Sf9 cells indicated that trehalose transport is preferred over glucose. *HaST46* expression was found to be modulated tissue-specific manner in response to dietary trehalose availability. Furthermore, trehalose synthesis genes were found to be downregulated in the case of a 50 mM trehalose diet. This suggests that a potential increase in exogenous trehalose uptake may attenuate its endogenous synthesis. *HaST46* overexpression and silencing lead to altered trehalose levels in the insect, while also having impact on trehalose metabolizing enzymes. Overall, our findings reveal the role of gut predominant sugar transporter, HaST46, in metabolic fine-tuning between exogenous trehalose uptake and endogenous synthesis.

## Introduction

Insects exhibit a remarkable metabolic adaptation that contributes to their ecological success (Terblanche and Lehmann 2025). One of the key reasons for their extensive adaptability is their metabolic dynamics and variability in response to different stimuli. As carbohydrates are primary energy source for fueling flight, development, reproduction, diapause, and stress tolerance, modulation in their level plays critical role in insect survival (Thorat et al. 2012; Feofilova et al. 2014). Specifically under stress conditions, insects precisely regulate their energy acquisition, storage, and mobilization (Zhang et al. 2019). Among different sugars, trehalose acts as predominant hemolymph sugar, fulfilling both structural and metabolic functions (Murphy and Wyatt 1965; Becker et al. 1996; Shukla et al. 2015; Matsushita and Nishimura 2020). Trehalose metabolism and distribution are essential for insect growth and adaptability (Tellis et al. 2023b). Trehalose is primarily synthesized in the fat body through a conserved pathway involving *trehalose-6-phosphate synthase* (*TPS*) and *trehalose-6-phosphate phosphatase* (*TPP*) (Murphy and Wyatt 1965; Becker et al. 1996). Following synthesis, trehalose is transported into the hemolymph and then distributed to the target tissues as per the energy demand through sugar (STs) and trehalose transporters (TRETs) (Tellis et al. 2023a).

Insects can ingest different foods to obtain complementary nutrients during nutritional stress (Raubenheimer and Jones 2006; Koyama et al. 2020). They further optimize energy utilization through metabolic balancing, enabling adaptation to diverse dietary conditions (Chown and Gaston 1999; Smith et al. 2018). Trehalose synthesis requires a continuous supply of ATP and carbon skeletons from glycolysis, glycogenolysis, or gluconeogenesis (Miyamoto and Amrein 2017). Depending on resource availability, insect may switch in between nutrient absorption or biosynthesis or differential distribution (Izadi 2025). Hemolymph trehalose levels range from approximately 5 to 50 mM, which is maintained by its continuous biosynthesis, degradation, and distribution (Murphy and Wyatt 1965; Kikawada et al. 2007; Al Hinai et al. 2025). TRETs serve as key molecules in regulating metabolic trade-offs under controlled and stressed conditions (Leyria et al. 2020). The midgut is the primary site of sugar and nutrient absorption, detoxification, and enzymatic conversions (Huang et al. 2015). To meet the metabolic demand, insects rely on sugar absorption or distribution from the gut tissues by sugar transporters such as TRETs, sodium-glucose co-transporters (SGLTs), and facilitative glucose transporters (GLUTs) (Chaudhari et al. 2025).

Phytophagous insects such as *Helicoverpa armigera* can acquire trehalose directly from the diet (Shukla et al. 2015; Tellis et al. 2023b). In *H. armigera*, trehalose-specific sugar transporters (*HaSTs*) showed diversity and spatio-temporal expression patterns (Tellis et al. 2023a). In dipteran and hymenopteran insects, glucose absorption is primarily mediated by SGLTs and GLUTs (Wang et al. 2016). Sugar transporter activity is closely linked to the insulin and TOR signaling pathways, facilitating metabolic plasticity by coupling nutrient availability with growth regulation (Riera et al. 2016). This critical trait allows insects to cope with environmental stressors, shift between trophic modes, and optimize energy expenditure during metamorphosis or overwintering (Danks 2007). The interplay between trehalose metabolism and other physiological systems, including neuroendocrine signaling, symbiotic relationships, and hormonal regulation, facilitates nutrient sensing, energy homeostasis, and ecological adaptability (Tellis et al. 2023b).

Despite advances in understanding trehalose metabolism within insects, the STs’ role and dynamics in response to dietary trehalose remain poorly understood. *H. armigera* serve as valuable models for metabolic studies due to their high dietary adaptability, rapid development, and stress resilience (Riaz et al. 2021). Elucidating role STs in regulation of trehalose metabolism in response to diet offers insights into the metabolic homeostasis and its impact on physiological processes (Dawkar et al. 2016; Zhou et al. 2023; Guan et al. 2024; Tian et al. 2025). Here, we shed light on the functional role of the trehalose transporter, *HaST46*, in mediating the metabolic balance between trehalose's exogenous uptake and endogenous synthesis. Through differential gene expression analysis, quantitative real-time PCR (qRT-PCR), liquid chromatography–mass spectrometry (LC-MS)–based metabolomics, enzyme activity assays, and genetic manipulation, we investigated how dietary trehalose influences *HaSTs* dynamics and activity, mainly in the gut. This study advances the knowledge of sugar transport, metabolic homeostasis. and nutritional physiology.

## Materials and methods

### Differential gene expression and proteomic signature analysis

Publicly available *H. armigera* gut transcriptomic and proteomic datasets (Ioannidis et al. 2022) were used for expression profiling of *HaSTs*. In that study, transcriptome sequencing was performed on whole midguts from artificial diet-fed L2, L3, and L4 larvae along with their corresponding carcass samples. The midgut portion of these samples was also analyzed via proteomics. For L5 larvae, RNA-seq and complementary proteomics data were generated across 5 gut compartments such as foregut (FG), anterior midgut (AMG), middle midgut (MMG), posterior midgut (PMG), and hindgut (HG), and for both plant-fed and artificial diet-fed larvae with these available datasets, we evaluated gut-specific expression and proteomic occurrence of the chosen putative trehalose transporters (*HaSTs*) based on our previous study (Tellis et al. 2023a). For transcriptomics, raw fragments per kilobase of transcript per million mapped reads (FPKM) values were extracted, log₂(x + 1) transformed, and row-wise Z-score normalized before hierarchical clustering using Euclidean distance and complete linkage in the pheatmap package (R v4.x). For proteomics, the absence or presence of HaSTs in the proteomics set was denoted in binary form. Heatmaps were generated using TBtools 1.09 (Chen et al. 2020) to visualize stage- and tissue-specific expression patterns. For bar plots, mean FPKM values ± SEM were calculated across biological replicates.

### *HaST46* overexpression in Sf9 cells

For overexpression, *HaST46* was cloned into the pIBV5 vector and sequence-confirmed. Sf9 cells (4 × 10^6^) were seeded in a T25 cell culture flask (Corning, NY, USA) with 1 mL serum-free SF900 II SFM media (Thermo Scientific, Waltham, MA, USA) and allowed to adhere by incubating for 30 min at 27 °C. For transfection, 1.5 µg of either the EGFP_pIBV5 control plasmid or the *HaST46-pIBV5* plasmid was diluted in 100 µL SF-900 II SFM (without antibiotics). Separately, 8 µL Cellfectin II reagent (Gibco, Waltham, MA, USA) was diluted in 100 µL Sf-900 II SFM and incubated for 30 min. The DNA and lipid solutions were combined (total 200 µL) and incubated for an additional 30 min to allow complex formation. The transfection mix was added dropwise while gently rocking the flask for an even distribution. Flasks were incubated for 5 to 7 h at 27 °C. After the incubation, the media was removed, and fresh Sf-900 II SFM containing blasticidin (Gibco, Waltham, MA, USA) antibiotic was added and incubated for 48 h. Overexpression was confirmed by qRT-PCR. For the sugar uptake assay, 5 and 10 mM concentrations of glucose and trehalose were added to the media, and the cells were incubated for varying time points (0, 5, and 10 min). Following incubation, the media and cells were collected for metabolite extraction and analysis. Sugar uptake was determined from changes in extracellular and intracellular sugar concentrations by metabolite profiling.

### Structural modeling and docking

In order to identify substrate-transport preference, SPOT analysis on the DeepMolecule server (https://www.deepmolecules.org/SPOT) was performed. Furthermore, the 3-dimensional structure of HaST46 was predicted using the Alphafold 3 server (https://alphafoldserver.com/) (Supplementary Information 1). Structural alignment of the best model was performed using PyMOL (The PyMOL Molecular Graphics System, Version 3.0 Schrödinger, LLC) using Human Glut1 (PDB ID: 4PYP) structure as reference. Binding site residues of Glut1 were used to predict the binding pocket of HaST46 and molecular docking of glucose and trehalose was performed using AutoDock Tool as described earlier (Barbole et al. 2024).

### Insect rearing

Larval populations of *H. armigera* were obtained from the ICAR-National Bureau of Agricultural Insect Resources (NBAIR), Bengaluru, India, and maintained on a chickpea-based artificial diet (AD) for multiple generations (Supplementary Information 2). Insects were reared at 25 ± 1 °C, 70 ± 10% relative humidity, and a 16:8 h light: dark photoperiod. Adult mating boxes, containing equal numbers of males and females, were provided with 10% (w/v) sucrose solution and 1% (w/v) vitamin E, with a muslin cloth for egg collection (Dawkar et al. 2016).

### Exogenous trehalose feeding and tissue collection for the bioassay

For exogenous trehalose feeding in the *H. armigera* bioassay, freshly moulted second instar larvae were fed AD supplemented with 10 mM, 50 mM, or 100 mM trehalose for 6 d. The components used were same to those described in the above table of artificial diet (AD), with the only modification being the addition of different trehalose concentration (10, 50 and 100 mM to the mixture for feeding assay. Then assess the impact on insect weight, nutritional indices efficiency of conversion of ingested food (ECI), efficiency of conversion of digested food (ECD), and approximate digestibility (AD) and expression of sugar transporter genes (*HaSTs*) (Supplementary Information 3). Three biological replicates (*n* = 10 larvae each) were used, and the samples were flash-frozen in liquid nitrogen and stored at −80 °C. For tissue collection, fifth instar larvae were starved for 1 to 2 h followed by washing with phosphate-buffered water and dissected under RNase-free conditions to collect hemolymph, gut tissues (FG, midgut, HG), and fat body. The samples were flash-frozen and stored at −80 °C.

### In vitro targeted putative sugar transporter gene *HaST46* silencing via dsRNA feeding

Silencing of *the HaST46* gene was performed using microbial-based dsRNA production. To generate dsRNA targeting *HaST46*, a 400-bp fragment was PCR amplified using Phusion DNA Polymerase (Thermo Fisher Scientific, Waltham, MA, USA) and a Proflex PCR machine (Thermo Fisher Scientific, Waltham, MA, USA) with primers (Supplementary Information 4). PCR conditions included 98 °C for 30 s, followed by 35 cycles of 98 °C for 10 s, 60 °C for 30 s, and 72 °C for 30 s, with a final extension at 72 °C for 10 min. The purified PCR product was cloned into the pGEM-T Easy vector (Promega Corporation, Madison, WI, USA), excised, and ligated into the L4440 vector using NotI and EcoRI. The ds*HaST46*-L4440 plasmid was transformed into *E. coli* HT115 competent cells. A single colony was cultured in a 5 mL primary culture with tetracycline and ampicillin. A 200 mL 2 × YT media with antibiotics was inoculated for secondary culture and induced with 1 mM isopropyl β-D-1-thiogalactopyranoside (IPTG) at an OD of 0.4. After 4 to 5 h at 37 °C, the cells were harvested, resuspended in nuclease-free water (OD 2.0), and mixed into the larval feed. A bioassay with 15-s instar larvae per treatment was conducted for 6 d using an empty L4440 vector as a control. The feed was replaced, and the parameters were recorded every alternate day. On day 6, 7 larvae per treatment were starved for 2 to 3 h, flash-frozen, and stored at −80 °C. Nutritional indices (ECI, ECD, ADI) and body weight changes were calculated. RNA was extracted using TRIzol, treated with RQ1 RNase-free DNase, and visualized on a 1.2% agarose gel to confirm ds*HaST46* production. qRT-PCR was performed to assess *HaST46* expression in the control and treated insects, using actin as a reference.

### Transient overexpression of *HaST46*

For overexpression, *HaST46* was cloned into the pIB/V5 vector (Thermo Fisher Scientific, Waltham, MA, USA) and sequence-verified. Larvae were starved for 3 h before injection. Further process was carried out according to the procedures described in the Supplementary Method 3 in Supplementary Information 4. A mixture of 1500 ng of each plasmid and Lipofectamine (Invitrogen, Thermo Fisher Scientific Inc., Waltham, MA, USA) (1:1 v/v), incubated for 30 min, was injected into the hemocoel between the fifth and seventh abdominal segments. The EGFP pIB/V5 plasmid served as the control (Barbole et al. 2024). Gene expression was quantified by qRT-PCR at 48 and 72 h post-injection, with 6 biological replicates (3 larvae per replicate). For tissue-specific expression analysis, the tissues were dissected 48 h after injection. Following injection, larvae were maintained on AD supplemented with 50 mM trehalose or control AD for 24 h, then flash-frozen, and stored at −80 °C (Supplementary Information 2).

### Quantitative real-time PCR (qRT-PCR) analysis

Total RNA was extracted from 80 to 100 mg of insect cells/tissues using the TRIzol reagent (Invitrogen, Waltham, MA, USA). To eliminate DNA contamination, RNA was treated with RQ1 RNase-free DNase. First-strand cDNA was synthesized from 5 µg RNA using oligo-dT primers and the High-Capacity cDNA Reverse Transcription Kit (Applied BioSystem, Foster, CA, USA), following the manufacturer's protocol. Gene expression was quantified using a 7500 Fast Real-Time PCR System (Applied Biosystems, Foster, CA, USA) with Takara TB Green Premix Ex Taq II. Primer list is provided in Supplementary Information 4. Expression dynamics by calculating fold change (2^−ΔΔCt), were visualized using GraphPad software. The expression of 5 *HaSTs*, *trehalose-6-phosphate synthase* (*TPS*), *trehalose-6-phosphate phosphatase* (*TPP*), and trehalase was assessed in trehalose-fed tissues, with the gut as the reference for fold change (2^−ΔΔCt) in fifth instar larvae. *HaSTs* expression was further evaluated in hemolymph, fat body, and silencing assays, using control expression as the reference.

### Metabolite analysis using LC-Orbitrap-MS

To extract metabolites, 80 to 100 mg of finely crushed sample powder was mixed with 500 µL of 80% MS-grade methanol to ensure efficient metabolite extraction (J.T. Baker, Center Valley, PA, USA). Then this mixture vortexed for 15 to 20 min at room temperature to enhance homogenization, then proceed for 20 min sonication to disrupt cellular content followed by centrifugation at 18,000 × g for 10 to 20 min to get clear supernatant. The supernatant stored in −80°C deep freezer to stabilize metabolites and prevent further degradation. The next day, samples were again centrifuged at high speed for 10 to 20 min to remove of residual particles and supernatant filtered through a 0.2 µm syringe filter to remove micro-particles that may interfere with detection. The LC method started with 2% B for the first 0.3 min that is the conditions the column for metabolite separation and for increase compound resolution solvent strength increased to 30% in the next 2 min. The B% was increased from 30% to 45% till 5 min and further increased to 98% to 7 min at which it was held for the next 50 s. The column was equilibrated to the initial ratio of solvents (98% A: 2% B) in the last 2 min to restores initial conditions for the next run. The MS data from 2 or 3 independent biological replicates and 2 technical replicates each were acquired in 5 GHz extended dynamic range (Supplementary Information 2).

### Enzymatic activity

The α,α-trehalase activity was determined by measuring the amount of glucose released from the hydrolysis of α,α-trehalose using the dinitrosalicylic acid (DNSA) reagent (Sigma-Aldrich, Merck KGaA, St. Louis, MO, USA). A premix containing buffer and equal volumes of crude sample extracts from the control and treatment groups were prepared. To this reaction, 150 μL of trehalose, i.e. 0.25%, was added. The reaction was allowed to hydrolyze the enzyme for 15 min at 37 °C. The reaction was then stopped with addition of 500 μL of DNSA reagent. The mixture was then placed in a boiling water bath for 5 min in order to enhance the detection of glucose through colorimetry. The quantitative measurement of glucose released during the trehalose hydrolysis was single at 540 nanometers. The absorbance of the measurement was retrieved at 540 nm. Under the defined conditions of the assay, 1 unit of trehalase activity was defined as the amount of enzyme liberated of 1μM of glucose per minute.

The enzyme activity of trehalose 6-phosphate phosphatase (TPP) was assessed by monitoring the release of inorganic phosphate (Pi) from the crude extract using the malachite-green reagent (Sigma-Aldrich, St. Louis, MO, USA). The assay was conducted following the modified protocol of (Klutts et al. 2003 ). The reaction was performed in a total volume of 100 µL with a final concentration of 1 mM trehalose 6-phosphate (Sigma-Aldrich, MA, USA), 2 mM MgCl₂ (Hi-Media, MS, India), 50 mM citrate-phosphate buffer (pH 4), and 35 μg of enzyme extract. The reaction mixture was then incubated at 57 °C for 35 min to allow for hydrolysis. The Klutts procedure was followed, wherein the reaction was first quenched by the addition of 2 volumes of malachite green 0.15% filtered, 1% ammonium molybdate (Hi-Media, MS, India), and 12.5% (v/v) concentrated HCl (Thomas Baker, MS, India). After spectrophotometric measurements of the absorbance at 630 nm, sediment was separated and color terbium titration was held. Color was developed for 5 to 7 min. It can be claimed that this composition effectively measures Pi release.

### Statistical analysis

All experiments contain 3 or more biological replicates. Data were expressed as mean ± SE using GraphPad Prism v8.0 (GraphPad Software, San Diego, CA, USA). The Student’s unpaired t-test was used to analyze statistical significance between groups. Asterisks indicate significant changes compared to control (*P < 0.05; **P < 0.01; ***P < 0.001, ****P < 0.0001).

## Results

### *HaST46* showed gut-specific expression at the transcript and protein levels

**Fig. 1.**
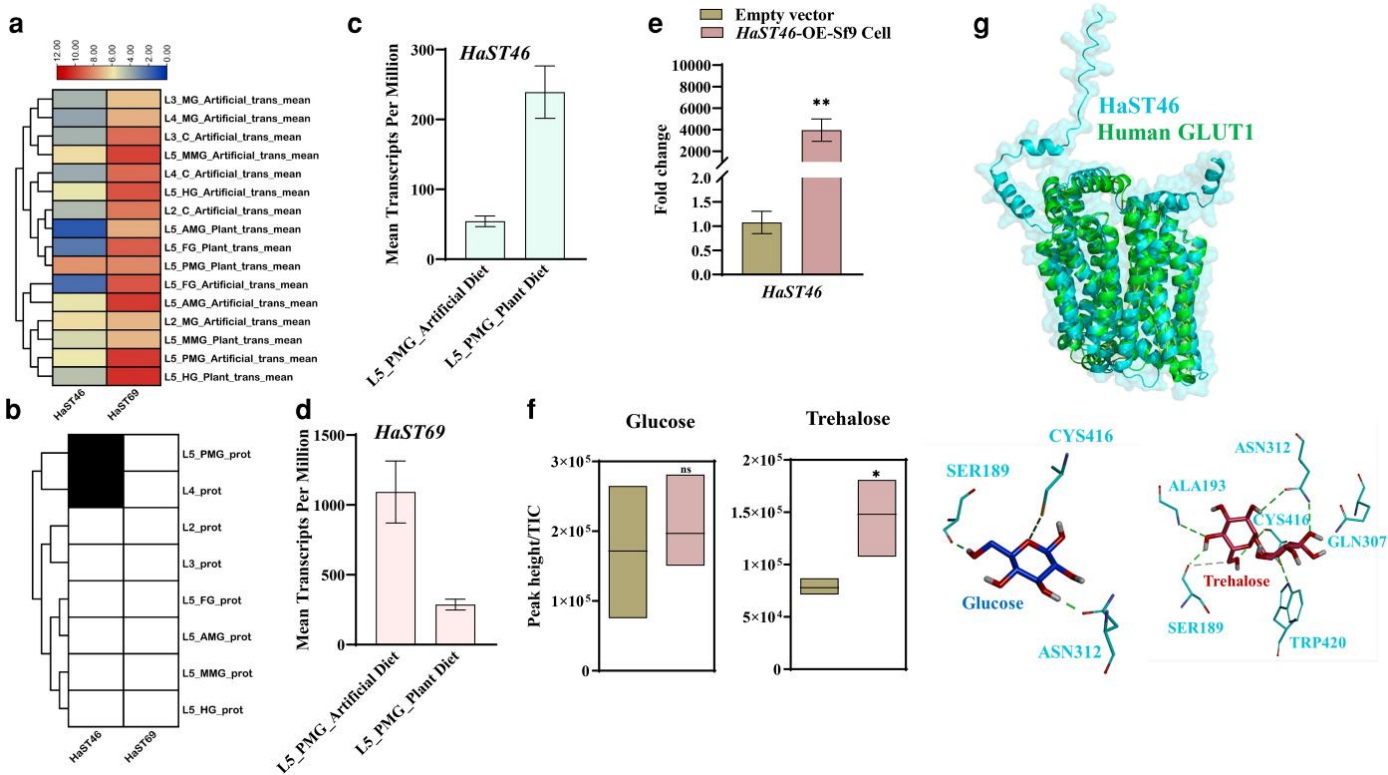
Spatial expression and functional characterization of *HaST46*. a) Effect of diet on putative trehalose transporters, i.e. *HaST46* and *HaST69* expression (transcript level; trans) pattern across 5 gut compartments: FG, AMG, MMG, PMG, and HG and carcass (C) in larvae fed on artificial diet (AD) and plant diet (PD). b) Protein abundance (protein level; prot) across larval stages (L2, L3, L4) and gut regions (FG, AMG, MMG, PMG, and HG). c and d) Expression (transcript) levels of *HaST46* and *HaST69* in the PMG under the 2 dietary regimes. This data analyzed using publicly available *H. armigera* transcriptomic and proteomic datasets (Ioannidis et al. 2022). Data represent mean ± SEM of biological replicates. Statistical significance was determined using an unpaired *t*-test (*P* < 0.05). e) Verification of *HaST46* overexpression by qRT-PCR. f) Quantification of D-glucose and trehalose in cell supernatants and lysates using LC-MS/MS after supplementation of the culture medium with glucose (5 mM, 10 mM) or trehalose (5 mM, 10 mM) at 0, 5, and 10 min intervals. All data are presented as mean ± SEM. Statistical analysis was performed using an unpaired *t*-test (*P* < 0.05). g) Structural conservation of HaST46 with human GLUT1 transporter. Molecular interaction of HaST46 with glucose and trehalose.

In our earlier published data we have described expression profiles of 14 *HaSTs*, out of which 5 *HaSTs* showed tissue dominant expression pattern (Tellis et al. 2023a ). *HaST46* and *HaST69* showed highest expression in gut and whole body, respectively (Fig. 1a). We analyzed the gut-specific expression and proteomic profiles of earlier studied 5 *HaSTs*, using publicly available *H. armigera* transcriptomic and proteomic datasets (Ioannidis et al. 2022). In the transcriptomics dataset, *HaST46* and *HaST69* showed high expression levels across larval stages (L2, L3, L4) and gut-region–specific expression in different gut regions, including FG, AMG, MMG, PMG, and HG in fifth instar (L5) larvae. *HaST46* expression peaked in the gut during the L4 larval stage and in the PMG of L5 larvae (Fig. 1a). In corroboration with transcript expression of *HaST46* and *HaST69* in the gut tissues, *HaST46* also showed proteomic level presence in the midgut (Fig. 1b). Given that feeding activity peaks during the fourth and fifth larval instars, elevated expression of the STs likely supports increased metabolic demand. Collectively, these data highlight that *HaST46* could be putatively involved in sugar absorption in PMG during the active feeder larval stage. Transcriptomics comparisons L5 larvae fed an artificial diet vs those fed a plant (cotton) diet (PD) showed *HaST46* expression was elevated in PD-fed larvae fed compared to AD, whereas *HaST69* exhibited the opposite trend (Fig. 1, c and d). In combination of proteomic presence, transcript abundance in gut and diet response increase expression of *HaST46* suggests its putative role in sugar absorption from feed in the gut. Furthermore, it was important to assess the sugar transport ability of HaST to establish their role in trehalose absorption from gut.

### HaST46 showed a preference toward trehalose transport

In order to understand sugar preference and transport ability we performed functional assays with 10 mM trehalose exposure to Sf9 cells overexpressing HaST46. LC-MS–based trehalose quantification of these cells revealed a significant increase in intracellular sugar accumulation in *HaST46*-expressing cells compared with the vector control (Fig. 1, e and f). *HaST46*-expressing cells accumulated ∼1.5-fold more trehalose relative to controls, while glucose uptake exhibited a moderate increase (∼1.2-fold), suggesting a trehalose preference of HaST46. In silico analysis using DeepMolecule (https://www.deepmolecules.org/SPOT) also suggested that HaST46 has more preference to trehalose (0.64) compared to glucose (0.58). Furthermore, the structural alignment of HaST46 with the human GLUT1 transporter (PDB: 4PYP) showed a high degree of conservation in transmembrane domain architecture with RMSD value of 3.67 Å (Fig. 1g). Molecular docking studies further revealed that the binding of trehalose (−6.2 kcal/mol) is lower compared to glucose (−4.7 kcal/mol) with HaST46, indicating trehalose structural preference over glucose for transportation. Molecular interaction studies showed CYS416 and ASN312 of HaST46 formed conserved hydrogen bonds with both glucose and trehalose. At the same time, TRP420 and ALA193 contributed additional interactions that might contribute to stabilize trehalose binding (Fig. 1g). These findings support that HaST46 may functions as a trehalose-preferred transporter, likely contributing to sugar uptake and homeostasis in *H. armigera.*

### Trehalose-rich diet feeding alters *HaST46* expression and trehalose metabolism genes

**Fig. 2.**
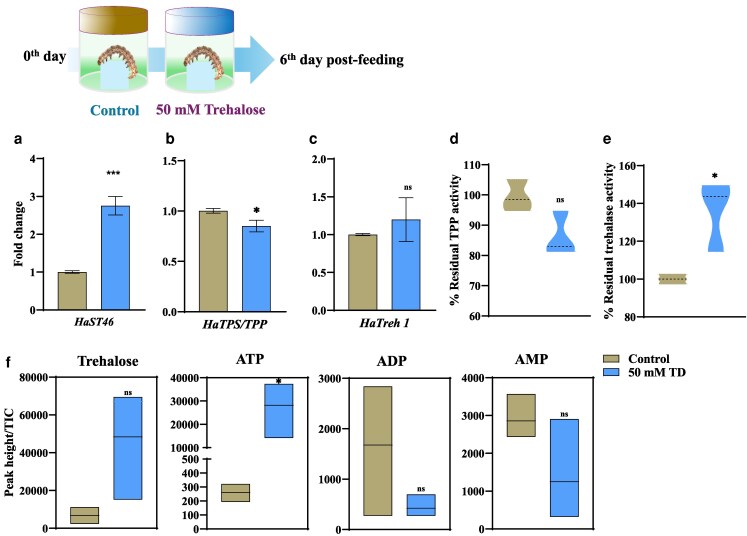
Gene expression and enzymatic activity upon exogenous trehalose feeding in *H. armigera*. Schematic diagram (upper panel): experimental design showing 2 groups of larvae (control vs trehalose-treated) and sampling timeline a to c) qRT-PCR analysis of *HaST46* and trehalose metabolism-related genes following dietary trehalose supplementation. Transcript abundance was normalized to reference genes and analyzed as a fold change (2^−ΔΔCt). d and e) In vivo activity of trehalase and trehalose-6-phosphate phosphatase (TPP) in larvae fed a 50 mM trehalose-enriched diet or an artificial diet. f) Quantification of trehalose, ATP, ADP, and AMP in whole larvae using LC-MS under the same dietary conditions. Data are presented as mean ± SEM, with statistical significance assessed by an unpaired *t*-test (*P* < 0.05).

To assess the effect of trehalose feeding on trehalose metabolism and transport, we performed a feeding bioassay with a trehalose-containing diet, followed by the insect's molecular response to the altered diet composition. Exogenous trehalose feeding showed normal growth and development (Supplementary Information 5). qRT-PCR analysis showed that feeding of trehalose-containing diet, induced changes in the HaSTs (Supplementary Information 5 and 6). *HaTPS/TPP*, *HaTreh1*and *HaST46* expression was altered upon trehalose feeding (Fig. 2a). Furthermore, *HaST46* was upregulated, and *HaTPS/TPP* was downregulated, while only *HaTreh1* exhibited a non-significant elevation in expression upon feeding a diet with 50 mM trehalose (Fig. 2, b and c). This alteration in the *HaTPS/TPP* expression level suggests a putative reduction in the energy-intensive endogenous trehalose biosynthesis and increased hydrolysis of exogenous trehalose for energy need. Notably, *HaST69* was downregulated in the 50 mM trehalose diet, whereas in 10 and 100 mM trehalose diet, it showed increased expression. This variable expression of different HaSTs might be due to their differential substrate specificity and spatio-temporal expression profile in response to dietary content (Supplementary Information 5). Residual TPP activity was reduced, while trehalase activity was high in larvae fed the 50 mM TD (Fig. 2, d and e). Metabolomic quantification revealed that the insect fed on the TD showed relatively greater accumulation compared to the control, although the changes were not statistically significant compared to the control (Fig. 2f). TD-fed insects showed an increase in ATP levels, whereas ADP and AMP levels remained unchanged, indicating ATP conservation (Fig. 2f). This evidence suggests a preference for hydrolytic metabolism under elevated dietary trehalose, which is likely to convert absorbed trehalose into glucose for systemic energy needs.

### *HaST46* showed gut-specific upregulation in response to trehalose feeding

**Fig. 3.**
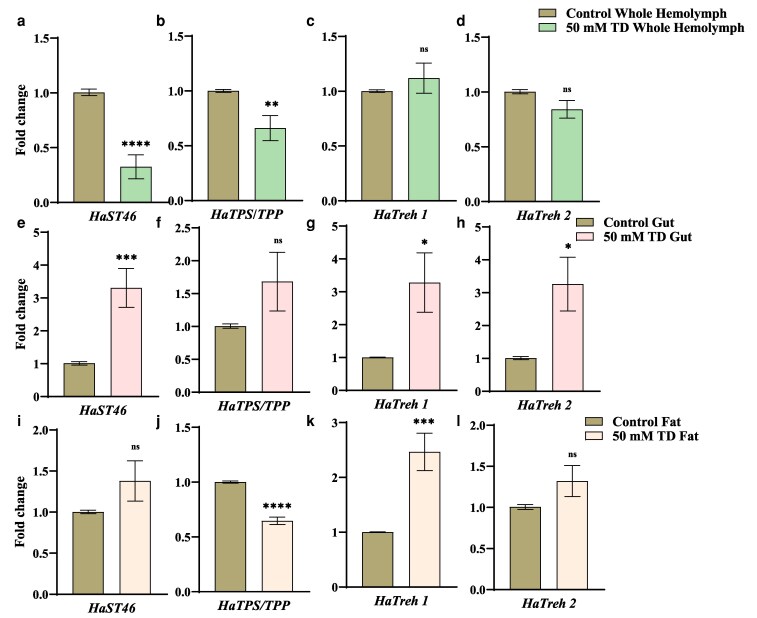
Tissue-specific expression of *HaST46* and trehalose metabolism genes following trehalose feeding. (a to l ) qRT-PCR analysis showing expression patterns in whole hemolymph, fat body, and gut tissues. Transcript levels were normalized to reference genes and expressed as fold change (2^^^−ΔΔCt) relative to control. Data represent mean ± SEM, with statistical differences determined by an unpaired *t*-test (*P* < 0.05).

Tissue-specific gene expression was further analyzed in 50 mM TD-fed insects to elucidate the dynamics of trehalose transport. The relative expression of *HaST46* and *HaTPP* was reduced in whole hemolymph (Fig. 3, a and b), while the expression of *HaTreh 1* and *HaTreh 2* was unaltered (Fig. 3, c and d). The gut showed a significant ∼3-fold increase in *HaST46* expression under a 50 mM TD (Fig. 3e), indicating an increase in the need to transport and use excess trehalose across the gut tissue. *HaTPS/TPP* expression was found to be unchanged, while *HaTreh 1* and *HaTreh 2* expressions were significantly high (Fig. 3, f to h). *HaST46* expression in the fat body (key tissue for trehalose synthesis) was found to be unchanged (Fig. 3i). Tissue-wise expression of other *HaSTs* also altered in response to trehalose feeding (Supplementary Information 5). Interestingly, the expression of *HaTPS/TPP* decreased significantly, indicating a reduction in endogenous trehalose biosynthesis (Fig. 3j). *HaTreh1* showed significantly higher expression in the fat body, while *HaTreh2* displayed a non-significant upregulation in the fat body of trehalose-fed insects (Fig. 3, k and l). These observations suggest that dietary trehalose alters trehalose transport and metabolism-related genes in tissue-specific manner.

### Metabolomic profiling of trehalose diet-fed insects reveals trehalose metabolism trade-off

LC-MS analysis was performed for the whole hemolymph of insects fed on an artificial diet (AD) and 50 mM TD diet to quantify systemic metabolic changes. There was increased trehalose level in TD-fed larvae, although this was not significant, particularly in the whole hemolymph (Supplementary Information 5). These results confirm that trehalose supplementation alters gene expression and endogenous trehalose levels in the presence of excessive dietary trehalose in *H. armigera.*

### *HaST46* knockdown induces compensatory upregulation of the trehalose anabolism TPS*/TPP* gene

**Fig. 4.**
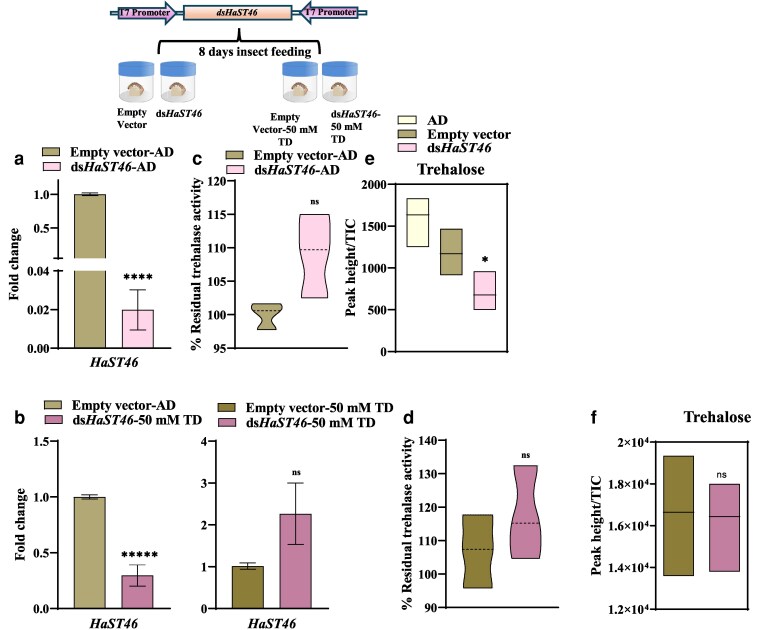
Silencing of *HaST46* in *H. armigera*. Schematic diagram (top): outline of dsRNA feeding protocol, control (empty vector), and treatment (dsRNA feeding). a and b) qRT-PCR analysis of *HaST46* expression in whole-body samples from control insects (empty L4440 vector) and dsRNA-fed insects, either fed on an artificial diet or an artificial diet supplemented with 50 mM trehalose. c and d) In vivo trehalase and TPP activities under the same conditions. e and f) Trehalose levels quantified in whole insects using LC-MS/MS. All values are mean ± SEM, with statistical analysis performed using an unpaired *t*-test (*P* < 0.05).

RNA interference (RNAi) was employed to silence gut-dominant *HaST46* expression (Supplementary Information 5). We achieved around >50% reduction in the *HaST46* transcript levels in *dsHaST46-*fed larvae compared with the empty vector (Fig. 4a) (Supplementary Information 5). Notably, larvae given the TD diet and treated with HaST46 dsRNA exhibited significantly elevated levels of *HaST46* transcripts compared to dsRNA-treated larvae on the AD control diet, suggesting that trehalose consumption can stimulate *HaST46* expression despite the presence of dsRNA-mediated knockdown (Fig. 4b, left panel) (Supplementary Information 5). Furthermore, trehalase activity was non-significantly elevated in TD-fed insects compared with AD-fed insects (Fig. 4, c and d). LC-MS analysis revealed a decrease in trehalose levels in the insect body upon silencing *HaST46* (Fig. 4e). However, upon trehalose feeding to *dsHaST46* insects, the trehalose level was almost the same as in the control (Fig. 4f). These findings highlight the potential role of *HaST46* in trehalose transport. The impact of *HaST46* silencing on nutritional efficiency was assessed by measuring nutritional indices such as ECI, ECD and, AD in both the control and silenced groups (Naseri et al. 2010). Nutritional indices were almost the same in both groups, such as the control and *HaST46* silenced insects (Supplementary Information 5).

The expression of other trehalose transporter genes (*HaST09*, *HaST29*, *HaST64*, and *HaST69*) was examined in *dsHaST46*-fed larvae to explore potential compensatory mechanisms. Silencing *HaST46* resulted in significant downregulation of these genes, suggesting overall impact due to reduced trehalose content upon silencing (Supplementary Information 5). *HaST09* and *HaST29* exhibited upregulation in exogenous trehalose, whereas *HaST69* exhibited downregulated in *dsHaST46* larvae fed on TD. However, *HaST64* and *HaTPS/TPP* remain unchanged (Supplementary Information 5). However, the functional significance of these changes requires further investigation (Supplementary Information 5). These results highlight the crucial role of HaST46 in regulating trehalose transport and metabolism.

### *HaST46* overexpression showed gut tissue prevalence, potentially influencing trehalose uptake

**Fig. 5.**
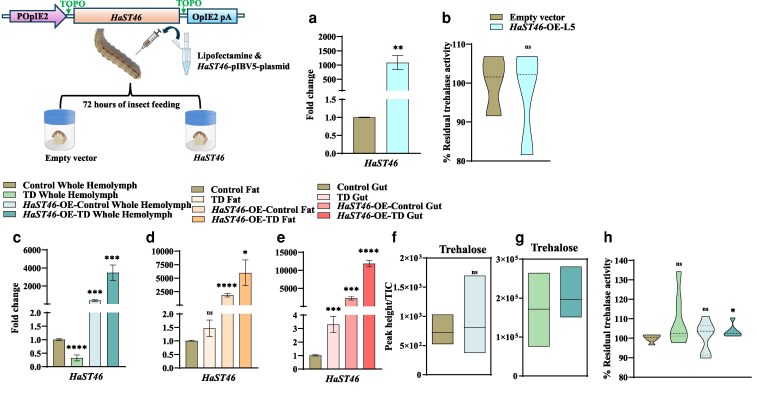
Overexpression of *HaST46* in fifth instar larvae of *H. armigera*. a) qRT-PCR expression of *HaST46* in the whole body. b) In vivo trehalase activity in whole-body samples following *HaST46* overexpression. c to e) Expression analysis of *HaST46* in the whole hemolymph, fat body, and gut tissues under 4 feeding conditions: artificial diet (AD), trehalose-rich diet (50 mMTD), *HaST46* overexpression with artificial diet, and *HaST46* overexpression with trehalose-rich diet (50 mM). Transcript levels were normalized to reference genes and expressed as fold change (2^−ΔΔCt). f to g) LC-MS/MS-based quantification of trehalose in whole hemolymph after *HaST46* overexpression. h) In vivo trehalase activity in whole hemolymph, fat body, and gut tissues samples following *HaST46* overexpression. Data are shown as mean ± SEM, with significance assessed using an unpaired *t*-test (*P* < 0.05).

To assess the effect of elevated *HaST46* transcript levels on trehalose metabolism and transport across tissues, we did transient overexpression of HaST46 in the whole body. Whole-body transient overexpression of *HaST46* was confirmed by qRT-PCR analysis, which showed a >1000-fold higher expression of *HaST46* in treated larvae compared with the control (Fig. 5a). *HaST46* overexpression in larvae did not significantly alter trehalase activity (Fig. 5b). Further, we observed the upregulation of *HaST46* in all 3 tissues (whole hemolymph, gut, and fat) of the *HaST46-OE* insects upon trehalose feeding compared with the control group (Fig. 5, c to e). However, there was a significant increase in the *HaST46* transcript level in a group of insects fed on a trehalose-rich diet in the gut, followed by whole hemolymph and fat. *HaST46* overexpression causes increased transcript levels in various tissues, with comparatively higher enrichment in the gut (Fig. 5, c to e). Trehalose levels in whole hemolymph were unchanged in TD-fed *HaST46*_OE insects (Fig. 5, f and g). At the same time, trehalase activity was also found to be high in TD-fed *HaST46*_OE insects in whole hemolymph (Fig. 5h). These results suggest that *HaST46* facilitates the uptake of dietary trehalose and plays a role in sugar homeostasis and energy balance in *H. armigera*.

## Discussion

Trehalose is key in energy homeostasis, functioning as a circulating sugar and substrate for rapid energy mobilization. Functional characterization of *HaST46* as a trehalose-preferred transporter with potentially localized to the PMG, principal site for nutrient uptake, highlights its putative function in dietary sugar absorption (Fig. 1, a and b) (Terra et al. 2023). Furthermore, the nutrition-responsive expression patterns of *HaST46* suggest that its expression likely depends on the diet composition and larval metabolic demand. The higher expression of *HaST46* in artificial or plant diet reflects an adaptive shift in response to trehalose availability in diet (Fig. 1, c and d). This adaptive plasticity aligns with earlier reports of the gut-specific modulation of nutrient transporters in response to dietary compositions in insects (Azzouz et al. 2005), underscoring the ecological and physiological relevance of nutrient transporter regulation.

The substrate preference of HaST46 for trehalose over glucose represents its potential functional specialization (Fig. 1, e and f). Despite structural conservation with GLUT1, HaST46 exhibits a preference for trehalose over glucose, suggesting an evolutionary divergence toward trehalose transport (Fig. 1g). This specificity likely enhances transport efficiency and optimizing trehalose handling in sugar-rich dietary contexts.

At the metabolic level, the changes in trehalose biosynthesis and degradation pathways in response to transporter activity reflect a coordinated homeostatic system. The downregulation of endogenous trehalose synthesis in the presence of abundant dietary trehalose suggests a resource-efficient switch that curtails ATP-intensive biosynthesis in favor of direct trehalose uptake (Fig. 2, a to e). An increased level of ATP compared to a low level of ADP or AMP suggests low demand for ATP hydrolysis in the exogenous supply of trehalose, highlighting energy conservation (Fig. 2f). This trade-off supports the notion that insects prioritize the least metabolically costly route to meet carbohydrate requirements, a trait advantageous for survival in fluctuating nutritional environments.

RNAi-mediated silencing reduces *HaST46* transcript abundance and triggers compensatory responses at both transcriptomic and enzymatic levels. The upregulation of *HaST46* in dsRNA-treated larvae on the trehalose diet suggests trehalose feeding could trigger a compensatory transcriptional response (feedback upregulation) of *HaST46* (Fig. 4, a and b). The upregulation of trehalose biosynthetic genes in *HaST46* knockdown suggests a potential compensation for reduced trehalose uptake by enhancing its endogenous production. Furthermore, *HaST46* overexpression indicated gut prevalent localization of elevated HaST46 molecules, supporting its preferred localization in the gut (Fig. 5, a and b). Together, these findings highlight role of *HaST46* in trehalose uptake in the gut and putative involvement in trehalose metabolism regulation in response to dietary trehalose. To elucidate the role of *HaST46* in trehalose metabolism and physiology by integrating RNAi and whole-body overexpressions; consequently, future tissue-specific overexpression to get insights into spatial role of HaST46 in trehalose transport. Given that trehalose metabolism is unique to insects and absent in vertebrates, *HaST46* offers a promising molecular target for the selective disruption of energy metabolism in lepidopteran pests. This study focuses on a specific lepidopteran species, potentially restricting the wider relevance of the identified trehalose transport dynamics among insects with different gut region and feeding behaviors. The temporal regulation of transporter expression during different developmental stages was not studied, leaving potential shifts in sugar uptake due to ontogeny unaddressed. While the present study offers an extensive overview of transporter expression throughout the gut, analyses targeting specific regions such as the FG, midgut, and HG may clarify the exact locations of trehalose absorption and reabsorption. Subsequent studies should prioritize clarifying the directional flow and mechanistic pathway of trehalose transport, especially in HaST46, which stands out as a significant transporter candidate. Site-directed mutagenesis along with in silico modeling and functional tests may uncover essential amino acid residues involved in substrate recognition, gating, and ion coupling, offering a molecular-level understanding of how HaST46 facilitates selective trehalose transport. These structure-function analyses would enhance our insight into the regulation of trehalose transport and could help formulate approaches to disrupt energy balance in lepidopteran pests.

## Conclusion

According to this study, HaST46 is an important gut-localized trehalose transporter that supports metabolic balance in *H. armigera*. It plays important role in dietary trehalose absorption as per internal metabolic demand by showing preference for trehalose and its regulated expression depending on the availability of dietary trehalose. Functional study, silencing and overexpression, showed that trehalose levels affected the expression of *HaST46* and trehalose metabolism genes, thus indicating a coordinated balance between external absorption and endogenous synthesis. These findings highlight the critical function of HaST46 in controlling trehalose metabolism and demonstrate the potential of this protein as a specific molecular target for disrupting energy regulation in lepidopteran pests. Future structural and functional aspects in depth will highlight the mechanistic importance of HaST46 in trehalose transport.

## Supplementary Material

jkaf303_Supplementary_Data

## Data Availability

Data are available in the figures of the article and its supplementary materials. All data supporting the findings of this study are publicly available. Any additional materials or information required for reproducing the results of this study are available from the corresponding author upon reasonable request. Supplemental material available at G3 online.
