# Supplementary material for: Mechanistic insights in the role of trehalose transporter in metabolic homeostasis in response to dietary trehalose
Source: G3 (Bethesda). 2025 Dec 30;16(2):jkaf303. doi: 10.1093/g3journal/jkaf303 (PMC12869065; doi:10.1093/g3journal/jkaf303)
Supplement: jkaf303_Supplementary_Data [file jkaf303_supplementary_data.zip › Supplementary_Information_S1_G3-2025-406339.docx]

***HaST09***

**>evm.model.scaffold_139.12 gene=HaOG203721_tret09**

ATGAACAAAGGAAGACTTAGGCAGTGTCATGCAGCCATGGCTGGCGGCTTCGGGTCGCTGGTCATGGGCGCTCTCAACGTCTGGCCCTCCTACACTTCAGAGCTCTACGCGTCCAACACCACTACTCCCCTATCTGCGCCTATGACGAAAGGTGAAGAGGCTTTGTTAGGGAGCCTGCCTTCGCTAGGAGCGATTCTCGGCTCAGCTGTAGCTGGGACACTGATCAATATGTTCGGGAGACGAAATGGTGGTGTTATATTGTCTTTGCCTTGTGTGATGTCCTGGGCAATAATAGGGGTAGCGAGTTCTATGAACCTGATTCTGGCAGCTAGATTCTTGTCCGGTATCACGGGAGGCAGCTTTCTAGTACTGACGCCTATCTTCATATCAGAAGTGGCTGAAGACGCTATTCGCGGAGGCTTAGCTTCAGCATCAATATTCCTATACTGCACGGGCACTCTACTCTCGTACATCGTGGGCTGGTGCCTCACGTACAGGACTATCATCTGGCTTCACTTGTTACTCAGCGTGTTGTGTACTCTACTTATACTGCTGGTGGTTGAAAGTCCCGTGTATCTGCTGCGGCAGAAGAGGGAAGAGGACGCAAGAGAAGCGATAGCGAAATACAGAGGAGTTCCAGTATCGTCAATGGTTGTGCTCGATGAACTGACGCGGATGAAGCAACAGATCATGCCGGCTGTCGAACTTGTGTCTATTACAGATATGGATCCCAAAGCAGAAGAAGCTGAAAAGCAGAAACTAAATAATGAAGAAGAGATCGTCCAAGAACAGCCTAAATCGATGTCACCGATCAAGTTACTGTTCGTATCACCGGTATCGCGGCAGGCGTTCATAGTTGTAATGACAGTCATCACGATGCAGGTATTCATGGGCATAGTTCCCGTACAAGTGTACGCTAAGACAGTGTTCACGGAGACAGACCCCAGCAAGTCAGACCTCTATACCGTCGTGTTTGCTGTCATACAGTTCTTTGGAGCTTTGACCTCTTCGCTTGTAGCTGATAAGGCTGGTAGACGGATTCTCATCATCATCTCGTCAATCCTCGTATTCCTCTGCATGGTGTCCCTCGGCTTCCTCCTGCAGACGCGCATGGCGCCGGCCTGGCTGACGGTGGTGCTGCTGATGCTGTACTGCTTCTCATTCCTCATAGGAGCTGGAAGTATACCCTATGTGTTGTTGGCTGAGGTCTTTGAGTCACAGGTCCAAGGCCTAGGTTCAATGATCGTCATAGAATGGGTTTGGTTCCTCAACTTCTTTATTCTAGGTATCTTCCCATATATGTTGAGTGCTCTCAAAATCCACGGGGCCTTCTATTTCTTTGGTGCCATGAGCCTTCTAAACGCAATATTAGCGTTTATACTAGTGCCGGAAACTAAGGGACTGTCAAATGCACAAATTCAGGATCTTTTTAGATCAAGAAAGAAGAATTAG

***HaST29***

**>evm.model.scaffold_227.8 gene=HaOG207130_tret29**

ATGGATTCGTATTTGAAGACACAGATTTTAATAGTGGCCTGTGTAAACATAGGCCAGTTCATAGACGGCTACAGTGTTGGCTGGTCAGCTCCCATCATCCCCAAGTTACAGGATCCTCAGCAGTCACCCCTGCCTGAGGTAGTGACTGACTTCCAGGTGGCCTGTATAGGATCCTTGCTGTATATTGGAGCTATAGTTGGTCCATATATCCCAAGCTACCTATCCAATGTAATAGGTCGGAAACCGTGCCTCCTACTAGGCGGGCTACTGAACCTTCTCGCCATCATCCTCATCATCACCACTCGTAACATCGCCATGGTGTATGCTGTTAGAATCATCAGCGGATTGGGGATGGGGATGGTGACAGTCAGTAACTTGGTGTACATTGGGGAGATTGCGTCTACAAACATCCGTGGCATACTCCTAACATCGACATCTATTGTTGGCATATTCGGGACGCTAGCAGCCTACAGTATAGGACCTTATGTGTCCTATGAAGCAACTGGTTATATAGCTCTGGTTATTAATATAGTTCATGTGATCGGTATATTGTTTATACCGGAGTCTCCTGTGTATTATGCTTTGAAAGGTAAAGAAACAGAAACCAAATCAACACTCCGACTTCTCGGCCGACTGGATGACTTAGAAAACGTCTTCGAGTCAGTTCAAGACTTGGACCCTAATGATGGCCACAGTTGGAAGTCATGGGTGAAGATATTCACTGTCAAGTCCAATAGAAGGTCCCTGATCATCACGTTTAGTCTGCTGACTCTTCAGCAGATGAGCGGAGTGGCAGCTGTGCTGTTTTTTGTGACTACCATATTTCAGCTGGCTGGGTCTTCGATACGGCCAGATCTAGCCACAATCCTGGTAGGTGCAACACGTCTTCTATCTAGTTTGATAGCACCAACGCTGGTGGAGAGAGCTGGCAGAAGAATCTTGCTTCTAACTTCCACTGCATTTTGTGCTATCAGCTTGTTTATCCTCGGCACATATTTCTATTTGGATCGGATACAGAGTTCCATCATTTCGGACATCAGATGGCTGCCGCTCATGGCTCTAATTATGTACTTCTTCTCTTATGAAAGTGGTTTCGGCACAATGCCCTTCGCCCTCGTCGGCGAGATGTTCAAGGGCAACGCCAGAAGTCCTGGCTCCGCCATCTCCATGACCACCGCTTGGCTCATCGGCTTCCTCATCGCCACCAGCTTCAACACCATGCTGAACAGCATCGGCAGTGACGTCACGTTCCTCGTATTCTCTCTCTCCTGTGTCCTGGCTTGCTTGTTCACTTACAAGTTTGTTCCTGAGACTAAGGGGAGGACTTTGAGTGAGATTCAGCAGATTTTGAGTGGGTGA

***HaST46***

**>evm.model.scaffold_34.66 gene=HaOG210281_Tret46**

ATGTGTGATGCAGTGTCACGGAAACATTCTGAGATGGGTGACGGAACAGTGCCAGAGTTAGAGAAAATCCATAAAAATGGAACAACGGACAATGGGAAAGAACTTAGTGCTTCTAATGATGAATACCTCAATAACCAACGATCCCCGTTTAGGAGACAGGCTATCATCTCATTTGGTGTGTTCATGCTCACCCTCGGGGCTGGTGCCACGTCAGGCATCTCTGCCATCTTAATTCCACAACTACAACATGCAAAGGGGAAAAAAGCCTTCTCAGTAGAATTGGTATCATGGGTAGCTGCCATATCATCGTTGGCTCTCTTCTTCGGTAACCTGATGTCGGGATATTTGATGGATAGACTTGGAAGAAGAATGTCTAGTCTACTTCTGGCGGGCACATTTGTGGCTGGCTGGTTGATTATTGGGTTCTCAAATGACCTCATGTTCCTAATCTTAATAGGAAGATTTATCACGGGGTTATGTCAAGGGTGGCTTGGCCCCCTTGGCCCAGTCTACGTAGGAGAATTTAGTAGTCCTGCTTACAGAGGACTGTTCTTAGCAGCTTTATCTTTAGGAATAGCAGTGGGTGTTTTCATGTCACATTTATTTGGCACTTTCCTGCATTGGAGTATATCGTCCTTGCTTTGTGGATTATTCCCACTGATTGGTGGTGTTATACTTTATTACGCACCAGAATCACCTTCGTGGTTAGCGTCTAAACAGCGAATTGATGAATGCATAATCTCGTACCAATGGTACAGAGGAAACAGTGCAGCTATGAAAACTGAACTTGATAAAATGATTGCTGATATTTCAGCGAAAGGCAATAATCAGAGTAAATTGCAAATTATAGCAGCGAACATAAAGAAGCCAGAGTTTTATAAACCATTAGGTATTATGACGACCTTCTTCGTTATAACGCAACTTTCTGGTGTCAACGTTATTTGTGCGTACACTACAGAGATGATGAAGGAACTCATTGGTAGTGGTTCGACTAGTTCACACGCCTACGCCGCTATGTTAAGTATAGACGTGTTGCGATGCGTGTCACTCGGCGCTGCTTGCATTATGCTCAGGAGATCAGGTAGAAGGCCCATGGCTATATTCAGTGGAGTATTCACATCGTTATCTTTAATTTCACTAGCTTTGTACCTGTATCTCAACGACTCTGGTGTCATCCATCACATATCACCATTCATTTCATTAGGTTTAATGGCATTCTACATAGTAGTATCCAATTTGGGAATATGTCCACTGCCATGGAATATGGTTGGTGAACTATTTGCCGTCGAAACTAAAGGAGTGTGTTCAGGCATTAGTGTCATGATGACTTCTGTGGCTTTCTTCGGAGTAGTGAAGACGGCGCCGTCCATGTTTAGAAGTATAGGTCACCATGGAACGTATCTCTTCTACGGGTTATCCACCCTGTGTGGTACCATATTCTTATACTTATGTTTACCCGAGACAAAAGACAAAACTTTGTTACAAATAGAAGAACACTTTAGGTATGGCAAGAAAAAGAATGATAGCAAAGAAACAGATAATATTTAA

***HaST46*_ds-sequence**

TGAGATGGGTGACGGAACAGTGCCAGAGTTAGAGAAAATCCATAAAAATGGAACAACGGACAATGGGAAAGAACTTAGTGCTTCTAATGATGAATACCTCAATAACCAACGATCCCCGTTTAGGAGACAGGCTATCATCTCATTTGGTGTGTTCATGCTCACCCTCGGGGCTGGTGCCACGTCAGGCATCTCTGCCATCTTAATTCCACAACTACAACATGCAAAGGGGAAAAAAGCCTTCTCAGTAGAATTGGTATCATGGGTAGCTGCCATATCATCGTTGGCTCTCTTCTTCGGTAACCTGATGTCGGGATATTTGATGGATAGACTTGGAAGAAGAATGTCTAGTCTACTTCTGGCGGGCACATTTGTGGCTGGCTGGTTGATTATTGGGTTCTCA

***HaST64***

**>evm.model.scaffold_70.36 gene=HaOG215283_tret64**

ATGAGTTTCAATAAAAACAACCCCAACGCCATGGGGAAGATCATGGGCTACATCAAGCAGCTCTCCACCGAAGTGGGTGGGAGCGAGCAAACTCGCCGAGGTCAAGGCGACGAGGAGAGGTTGTACCGGACCCGAGGGCCCAAGTACTCTAGAGTCCCATCAAGACCGACGCTTTCTGCTTCTACGACCTGTACCTCTCTGGCAGAATCTTGTGGGTCCCAAGGGACATTGGTGCCTAACTACGCAACAATCCCAGAAACAGTTTCCACTGAAAGCAGCAGTGAAGACGAGCAGGACTCATTCGAGAACACCCGTCGCCATTTCCAACAACTTCGGCAGATCAGTCTAGGAAACGAGTTTAAGTACAAGATGGAGATGGAAATAAAGAGTGCGAAGGAGGAGAATTTGAGAAATTCGATTCCTTTTGTCAAACAACTGAGCACTGACAGCAGTAAAGTAAAACCGGACTATGCAATCAACGGGGATACTCCACCATATGCTCCAACAACCCAACGACTATTTCTGTGGACACAACTTTTGGCCGCATTCGCTGTGTCTATGGGTTCGTTGATTGTCGGCTTCTCGTCCGGCTATACTTCTCCTGCATTGATAAGCATGAACTCTACGCTTCACATGACTAAAGAAGAGTCAACATGGGTCGGCGGTCTTATGCCTCTGGCTGCATTGGTTGGTGGAGTCGCAGGAGGACCTCTGATAGAATGCATTGGAAGACGATGGACCATAATGGGAATGGCTTTACCATTCTTCCTCGGCTGGATGCTCATAGCAACTGCGTCAAACGTGCTAATGGTGTTCGCTGGAAGAGTTTTCTGCGGAGTGTGTGTCGGAATAGTCTCCCTGGCATTCCCAGTTTACCTCGGTGAAACGCTACAGCCCGAAGTACGAGGTGCGTTTGGATTGTTGCCTACTGCCTTTGGTAACACTGGAATACTTTTATCATTCTTTGTGGGAAGCTACCTTGACTGGTCGAAACTAGCATTCTTTGGAGCTGCATTACCGGTACCATTCTTCCTGCTCATGCTGCTTACGCCTGAAACCCCACGCTGGTTTGTGTCCAAAGGACGCCCTGAAGATGCTCGTAAAGCGCTTCAATGGCTTCGAGGAAAAAATACAAACGTTGACAAGGAAATGAAGGATCTTACACGTACACAGGCTGATTCGGATAGAACAGGAGGAAATGCTTTCAGACAACTTTTTACTCTTAAATACATGCCCGCTGTCCTTATTTCTCTTGGATTAATGTTGTTCCAACAGTTAAGTGGTATTAATGCAGTAATTTTCTACGCCGCGTCAATCTTCAAAATGTCTGGAAGCACTGTTGACGAAAACTTATCTAGTATCATAATTGGAATCGTCAACTTTGTTTCTACATTTATTGCCACAGCTATCATTGACCGCTTGGGACGTAAAATGTTGTTATACATTTCCTCAGTTTCTATGATAGTTACTCTAGTTTCACTGGGAGCTTACTTTTATGTGATGGATTCAGGAGTTGATGTCACGGCTTTTGGATGGTTGCCACTTGCTTGTCTTGTCATTTATGTATTGGGATTCTCTATTGGCTTTGGACCCATCCCGTGGCTCATGTTAGGTGAAATTCTACCATCCAAAATCCGTGGCACAGCTGCATCTCTTGCGACTGGATTCAACTGGACGTGTACCTTCATTGTCACTAAAACTTTCCACAATATCATCGACGCCATTCATATGTACGGTACAGTGTGGCTGTTTGCTGTCATTTGTTTAATTGGGCTGTTTTTCGTAATATTCTTTGTCCCTGAGACTCGAGGTAAAAGTTTAGAGGAGATTGAAAGGAAATTAACAGGGGGTTCACGAAGAGTGCGGCATATTAGCAGCAGTAAGCAACCACAAAATGGCTGTTAA

***HaST69***

**>evm.model.scaffold_8.129 gene=HaOG215993_tret69**

ATGAGGCTCACGCGGCGCAGGTGGGCAGAACTCCAAATATTCCGAACAAAAGCTACGCTTATCACCGCTACAGCGGGTACCTGCTACGGGTGGCCCTCACCTACTCTACCGTACCTACTATCCGAAGAGAGTTCAATCAAAACAACAGCTGACGAGGGATCATGGATAGTCTCGATAATGATCCTGTGCTCCGCGTTGACGCCTGTTCCCTCCGCCTACTTCGCCGACCGGTTCGGCAGGAAGACCACGCTACTCCTCGGTGCGGTGCCGTTCATCCTGGGTTGGGTGCTGGTCATCGTGGCCAACTCCGTCGCTCTGCTCTACGTGGCGCGGATGTTCTCCGGCTTGGGCTATGGAATTGTCTACACAGTTGCTCCAATGTACACAGGAGAAATCGCTACCAATCAAGTTCGAGGAGCCCTCTCCACACTCATCACGTTAATGAATAAAGTCGGAATTCTTGCCCAGTACTGCATCGGTCCGTTCGTCTCGATGCAAACCCTCGCTGCCATCAACTTGATCCTGCCCGTCACATTTGTCATCACCTTCATCTTTTTGCCAGAATCTCCTTACTACTACTTAAAATTTGAGCGAAGTGAGAGAGCCGAGCGCTCACTCAGGAATCTACGCAGTGGCGACATTAGAACTGAACTCAAAAGTATAGAACTGAACGTTCAAGAAGACATGAAGAATAGAGGAACTTGGGGAGACTTGATCACTGAAGCTACCAACAGGAAAGCAATGTGGATTACGCTTGGTATATTCACGATACAGCAGCTATGTGGCAGTGCTGCTGTGGTGGCATACGCGCAGGTCATATTCAACTGCACGACCAGCCCAGTCACTCCAAACATTACTGGAGCAGAAAATGTTACCGCTTCCGCTTCTATTGAACCCTACCAAGAATCTATTATTCTTGGTTGTGTACAAGTAGCCACCTGCGTCCTGTCAGTAATACTCGTCGACCGTGTTGGTAGAAAGCCCCTTCTGTTGCTATCGGCTCTTGGAGTGGGCCTTATGAACGGCACAATTGGAACATACTTCTACTTCGACCATGTCAACAAAGAAGCCGTTGCACATCTTCACTGGATACCCCTCGCCGCTCTTCTCGTTTACATCGTTTGCTACGCCATCGGGTTGTCGACGGTACCCTACGTCATCATAGGAGAGATGTTCCCGACCAACGTCAAGTTGTACGCTTCCTGTATCGCTCACATCTACACCGGCGTCTCCATGTTCGCTGTTCAAAAACTATTCCAGGTGGTCAAAGACGCATATCAAATCTACACAGTATTCTGGGGATTCGCCACGTTCTCGCTGCTGGGGCTGGTGTTCATGCTGATCATGTTGCCGGAGACGAAGGGCAAGTCGTTCGCGAGCATCCAGGCGCAGCTCAAGCGGGAGGTGGCCCGCGACAACGCTAAGAAACTGGCCACCGTCGAATACTGA

**EU878265.1 (159-2639_cds) *Helicoverpa armigera* trehalose 6-phosphate synthase isoform I mRNA, complete cds, alternatively spliced**

**>mRNA_tps_aa(6-507)final_nt(174-1679)**

AGCAGTGCCAGTCGATCCGCGTGCAACAGCAAGGGAAGCATGATCGTTGTGTCGAACAGGTTGCCCTTCATCCTCAAGAGAAATGACAAGACTGGCGGTCTGGAGAGGAAAGCCAGTGCTGGTGGGTTGGTGACAGCAGTAGCTCCAGTGGTGATCCGTGGAGGCGGCATCTGGGTGGGATGGCCAGGCATACATCTGGATGACCCCAATGAAAAGATCCCGGAGTCAGACCCCAACGACAAGACCCCTACTGCCGGCTTGCTATCAGAGAAGATAGTCCCCGTGCACGCTGAGCCCAAACTCTTCGACAGCTACTACAATGGCTGCTGCAACGGTACTTTCTGGCCCCTCTTCCACTCCATGCCTGACCGAGCCACCTTCATCGCTGACCACTGGAGGGCATACATCAAGTGCAACGAGGAGTTCGCTGAGAAGACAGTGTATGCTCTGCATCTGCTCAAACAACAGAAGGGGAAGAATGGGACCTCTCCACCGATCGTGTGGGTCCATGATTACCATCTTATGTTAGCTGCTAACTGGATTAGACAGCGAGTTGAGGAAGATGACATAAAATGCAAGCTTGCGTTCTTTCTGCACATTCCTTTCCCCCCGTGGGACATATTCAGGCTGTTCCCATGGTCTGATGAAGTATTGCAGGGCATTCTTGGTTGTGACATGGTCGGATTCCACATAACTGACTACTGCCTGAACTTCATTGATTGTTGCCAAAGAAACTTAGGTTGTCGTGTGGACAGAAAGAATCTGCTAGTTGAATTGGGAGGTCGCACCATCTGTGTCCGACCGTTACCTATCGGAGTACCCTTCGACAGATTTGTCCAGTTGGCTCAAAACGCAAAGACAGTGCTCTCTACAAGCCAACAAATTATATTAGGAGTTGATAGACTGGATTATACCAAAGGATTAGTACATAGACTGAAAGCTTTCGAGAGATTACTGGAAAAGTATCCCGAGCACATCAAGAAAGTAATGCTGCTTCAGATCTCGGTGCCGTCAAGAACGGACGTCAAGGAATACCAGGACTTAAAGGAAGAGATGGATCAGCTGGTTGGAAGAATAAACGGAAGATTTACTACTCCAAACTGGTCACCTATTAGGTACATTTACGGATGCGTCGGCCAGGATGAACTAGCTGCCTTCTACCGCGATGCTGCAGTAGCCCTGGTTACACCTCTGCGAGATGGCATGAACCTCGTCGCTAAGGAGTTCGTAGCCTGTCAGATTAACAAGCCTCCAGGAGTGCTGATCGTGTCACCCTTCGCCGGTGCTGGAGAAATGATGCACGAAGCTCTCATCTGTAATCCGTATGAATTGGACGATGCTGCTGAAGTCATTCACAGGGCGCTGATAATGCCGGAAGATGAGCGCACAGTCCGTATGAACCACTTGAGAAGACGTGAGCAGCTCAATGATGTTGATAGCTGGATGAAGGCGTTCTTGAAAGCCATGGAC

TCTTTGGAAGAGGAGGCTGATGATATTGGTGCCACG

**>mRNA_tpp_aa(512-783)nt(1692-2507)final_nt(1681-2529)**

CCATGCAGCCTGTCACCATTGATGACTTCGATGAATATCTTTCTAAGTACATTGGCTACACACAAAAGCTGGCATTACTACTTGACTACGATGGTACTCTAGCCCCCATCGCGCCTCACCCTGACCTGGCAACCTTACCCTTGGAGACCAAGCATACTCTGCAGGGGCTGTCCAATATGTCCGATGTCTACATCGCCATCATCTCCGGCAGAAATGTCAACAACGTTAAGAATATGGTTGGCATTGAAGGCATCACGTACGCTGGTAACCATGGTCTGGAAATCCTGCACCCAGACGGCAACAAGTTCGTTCATCCCATGCCCATGGAGTTGCAGGACAAAGTCGTCGACCTGCTCAAGGCTTTGCAAGAACAGGTGTGCAAAGACGGAGCCTGGGTAGAGAACAAGGGAGCTCTCCTGACGTTCCACTACCGCGAGACGCCGGCTGACAAGCGGCCGGCGCTGGTGGAGCAAGCCCGCAAGCTGATCACGGCGGCTGGCTTCACGCCCGCGCCTGCCCACTGCGCCCTCGAGGCCAGGCCGCCCGTCGAGTGGGATAAGGGTCGTGCATCCATTTACATCTTGAGGACAGCGTTCGGTTTGGACTGGAGCGAAAGGATTAGGATTATCTATGCTGGTGATGACGTCACCGATGAAGACGCCATGTTGGCCCTCAAAGGTATGGCAGCTACATTCCGCATCGCTTCATCCCAAATCACGAAGACATCAGCTGAACGTCGTCTATCCTCCACGGGCTCAGTACTGGCCATGCTCAAATGGGTGGAACGTCACTTTTCCCGCCGCAAGCCGCGCGCCAACTCGTTGACGTACAAAAGCGCGCGAAAGGCCA

***Ha-trehalase-1***

**>ENA|KJ652557|KJ652557.1 *Helicoverpa armigera* soluble trehalase mRNA, complete cds.**

GCAGTCGTATCCGCGATCGCGACTCGTGCGCATGTAATCGATTATTCCGACGAAATGTCGAATCGATAAAACAATCGATAAACATTGATACGATCGTTTAGTTGTACCAGTGAAATAGAGTAATATTAGTTTTTGTTTATTAGTTTATTCGAGATTTTATCGATATATCGAGTTGTCTGGACGAGTTGTGATTTGTAAGCTGTGAAGTGAATGGTGTCCTTTTGTAAGATGCGAGAACTCCTGATCTTGTTGGCGGCCGCGGCTGGGCTGGCCAGCGCTGACCTGCCACTCACCTGCACCAAACCCGTCTACTGCAACAGCAACCTGCTCCATCAAATCCAAATGGCGAGGCTCTACAATGACTCCAAGACCTTCGTAGACCTTCAAATGAACTTCGATGAAAACAAAACTTTGACCGACTTCGAAACCTTTTTCAACCTTCATAACAAAAACCCGACTAAGGAACAGTTGATGGAATTCGTCAATGAATACTTTTCCAACGACAACGAACTGGAGCCATGGCAGCCAAAAGACTTCAGTGACAATCCAGCATTTCTTGCTAAAATAAAGGACGATGCGTTAAGGGAGTTTGGAAAAGGTATCAATAACATTTGGCCACTTTTGGCACGGAAAGTTAAAGCAGAGGTGTTCCAGAAGCCCGATCAATTTAGTTTAGTACCCCTGACTCATGGATTCATAATACCCGGTGGACGATTCAAGGAAATCTATTACTGGGACACTTTCTGGATCATTGAAGGTCTTTTGATAAGTGGAATGCAA

GAAACCGCTAAAGGAATGATTGAAAATCTCATTGAATTATTGAATTTATTTGGCCACATCCCTAATGGTAGCAGAGGGTATTACCAGCAACGTAGTCAACCTCCTATGTTAAATGCCATGGTGGCTACTTACTACATGTATACCAAAGATCTCGAATTCCTCAGAAATAACATCGCATACTTAGAAAAAGAATTGGACTTCTGGATGGATAATAGAGTGGTATCAGTTAACAGAGGAGGTAAAAATTATACGCTTCTTAGATACTATGCCCCAAGCAAAGGCCCTAGACCCGAATCGTATTATGAGGACTACAGCAACACTGAAGGTTTTTCGGAAGAAGACAGTACCAATTTCTGCATCGATATCAAAAGTGCGGCTGAGAGCGGGTGGGACTTCTCAACGCGTTGGTTCCTCATGCCAGACGGCAGTAACAATGGCACTTTAACTGATCTGCACACGCGGTACATCATACCCGTTGACTTAAACGCCATCTTCGCCGGAGCTGCCCAGTACGTGTCAAACTTCCACGCCCTCTTAAAGAACCCGCAAAAAGCTGCTAGGTACGGACAGCTAGCACAAACCTGGAGAGACAACATTCAGGCAGTGCTGTGGAACGATCAAGATGCGATGTGGTACGACTTCAATATTAGGGACAATTTACATCGCAGATACTACTACTCGTCTAACGCTGCGCCGCTATGGCAGAATGCCGTTAATCCAGATTTTCTGAAACTCAATGCTGACAGGATTTTGAAAGCTATCACTGAATCCGGAGGTGTAGACTTCCCCGGAGGTGTACCCACGTCGCTCATCAGGAGTGGAGAGCAGTGGGACTTCCCCAATGTGTGGCCTCCAGAGGTGAGCATCGAAGTCGCTGCTATTGAGAATATCGGGACGCCTGAGGCTATTACTTTGGCGCAGGAAGTAGCACAAACTTTCGTGAGGTCTTGTCACTGGGGCTTCCAGAAGTACAAGCAGATGTTTGAGAAGTACGATGCCGAGACGCCCGGCAGGTTCGGCGGTGGCGGTGAATATAATGTGCAGTTCGGTTTTGGTTGGAGTAACGGCGTCGTACTGGAATTTCTAAATAAATATGGGTCTCAGCTAACAGCCGACGACTCTAACAATACGAATAATAGTGCATGACTAGGCGTGGATATTGTGAACAATTTCCTAATTTTCATATCCACTATTAAAATACTTATAGCTTCCTACATTAGTACATAATATTTGAAGGTTGTGAACTTACGATTTTGACTAAATTAAGTGATTCTGACTAAAAACGGTGCCATCACTAATGAAATTTATATAAAAAAAAAATAAAACCGTGTGATGTCGGATAATGATAGAACATTTTACTCTACAACAGCGTTATACAGACAGGCTGTAAGAAACTCAGGTGCTTCAGACCAGGCAGAATATATTTCCCAATACTTAATTTAATAGAACAAAAAAAAAAAAAAAAAAAAAA

***>Ha-trehalase-2***

ATGGATCGGAGTCACTTGCCACCGACCTGTTCTAGCACCATCTATTGCCACGGGCCCCTACTAGACACGGTACAAATGGCGGGCCTGTACAACGACTCTAAGACCTTCGTGGATATGAAGCTCAAGCTGTCTGCCAACATCACCATGGAACACTTTCAGGAGATGATGGCCAGGACAGGTTCACACCCGACCAAGGCTGACATCCAGGAGTTTGTCAATCAGAACTTCGACCCTGGGGGCTCCGAGTTCGAAGACTGGCGGCCTACTGACTGGAAGGATAATCCTGCATTTCTGCAAAACATCAAGGATCCTCTGCTCCACCAATGGGCTGCAGAGCTGAACAGACTGTGGTTACAGCTTGGCAGGAAGATGAAGCCGCATGTGAAGAACAACCAGGATCTGTACTCTATTATCTACGTGGATAATCCGGTTATTG

TGCCTGGTGGTCGTTTCCGAGAGTTCTACTACTGGGACTCCTACTGGATCATCAAGGGTCTGCTTCTGTCCGAGATGAGGGCCACAGCTAAAGGCATGGTGTCAAACTTCATGGATATTGTGGAGAGGATCGGCTTCATTCCCAATGGGGGGAGGATATATTATGCTATGAGATCACAGCCCCCACTCCTAATCCCCATGGTGAAGATAATACTGGATGATATGGACGACCTGGAGTACTTGCGCCAACACATACACACCTTAGACAGAGAGTATGACTACTGGATGACTAACCATACTGTCGAGGTGGACCATAATGGGCATAGATACACGCTAGCGAGGTATTACGATCAGTCACAAGGACCCAGGCCTGAGAGTTACAAGGAAGACGTCGATGTGGCTAGACACTTTGACACAAATGACAAGAAAGAGGAGTTATACGCCGAGCTGAAGGCGGCTGCTGAGCCAGGATGGGACTTCTCATCCAGGTG

GTTCATACTCAATGGCACCAATAGAGGTAACCTAACAAACCTGAAGACCCGCTCCATTATCCCGGTGGACCTCAACGCCATCATGTGCTGGAACGCACAACTCCTGAGAGACTTCCACCTCAGGCTCGGCAATATCGATAAGGCGGAGTACTATAGGAACGTTCATGCGAGGTTCATGGATGCTATTGAACAGGTCCTATGGCACGAAGACGTAGGAGTCTGGCTAGACTACAGCCTGGAGTCGGGCAGACGCCGCGATTACTTCTACCCGTCAAACGTGTCGCCTCTATGGGCAGTCTGCTACGATCAGGCCAGAAAGGACTACTATGTCAACAGAGTTGTTAACTATCTGGATAAAGTTAAAGTGGACATTTTCGACGGCGGCATCCCAACAACTTTCGAACATTCTGGAGAGCAGTGGGACTACCCGAATGCCTGGCCGCCATTACAGTACATAGTGGTAATGGGCTTAGCTAATACTGGCCAGCCA

GAGGCTGTGAGACTGGCCAGCGAGATCGCTACGAAGTGGGTGCGTTCGAATTTCGAAGTTTGGAAACAGAAGACTGCTATGCTTGAAAAGTACGACGCGACAATTTTCGGCGGTCTCGGCGGAGGCGGCGAGTACGTTGTACAAACAGGCTTTGGTTGGACCAATGGCGTGATCATGGCCATGCTCAACAAATGGGGAGATACGCTTACTTCAGCGGACGCGTTCGGGACGGGCGTGACGGCTGACTCCGGTGCTGTGTACGGAGCGCATGTCGGCGCTAGCGGCGTGGCCACGGCTATTCTAGTAGTGCTCGCATCTTTGGCTGCGGGGACCCTTGGACTCATCGTATACCGAAAACGCAGGGACTACATCCGAGTATCAGGGGGCGAAGACTACAAATTGCTCTCCCGAAGACCTTAC
