# Supplementary material for: Mechanistic insights in the role of trehalose transporter in metabolic homeostasis in response to dietary trehalose
Source: G3 (Bethesda). 2025 Dec 30;16(2):jkaf303. doi: 10.1093/g3journal/jkaf303 (PMC12869065; doi:10.1093/g3journal/jkaf303)
Supplement: jkaf303_Supplementary_Data [file jkaf303_supplementary_data.zip › Supplementary_Legends_G3-2025-406339.docx]

**Supplementary Information Legends**

**Supplementary Information S1.** *H. armigera* *STs* gene sequences

**Supplementary Information S2.** General methods for real-time PCR, TPP and trehalase enzymatic activities. Artificial diet and trehalose diet components

**Supplementary Information S3.** Name of putative *HaSTs* and its corresponding gene id from NCBI gene database and scaffold id from transcriptome data

**Supplementary Information S4.** *HaSTs* primers used for real-time PCR analysis and silencing experiment

**Supplementary Information S5.** H. armigera growth phenotype, nutritional indices and HaST46 expression along with selected putative *HaSTs*, analysis upon dietary modulation, gene silencing and overexpression

**Supplementary Information S6.** All the neumerical raw data of real-time PCR, enzymatics assay and metabolomics quantification
